# MedMinas project: design and use of mixed methods in the evaluation of pharmaceutical services in primary health care in Minas Gerais, Brazil

**DOI:** 10.1186/s12874-022-01568-y

**Published:** 2022-03-27

**Authors:** Tatiana Chama Borges Luz, Noemia Urruth Leão Tavares, Ana Karine Sarvel de Castro, Isabela Cristina Marques, Elizabeth Moreira dos Santos, Betania Barros Cota

**Affiliations:** 1 GETESA (Grupo de Estudos Transdisciplinares em Tecnologias em Saúde e Ambiente), Rene Rachou Institute (IRR), Oswaldo Cruz Foundation (FIOCRUZ), 1715 Augusto de Lima Ave, Barro Preto, Belo Horizonte, Minas Gerais 30190-009 Brazil; 2 Strathclyde Institute of Pharmacy and Biomedical Sciences (SIPBS), University of Strathclyde, Glasgow, G4 0RE UK; 3 Department of Pharmacy, Faculty of Health Sciences, University of Brasília, Darcy Ribeiro University Campus, Asa Norte, Brasília, Distrito Federal 70910-90 Brazil; 4 LASER (Laboratório de Avaliação de Situações Endêmicas Regionais), National School of Public Health Sérgio Arouca (ENSP), Oswaldo Cruz Foundation (FIOCRUZ), 1480 Leopoldo Bulhões St, Manguinhos, Rio de Janeiro, 21041-210 Brazil

**Keywords:** Health Services Research, Mixed-Methods Study, Primary Health Care, Pharmaceutical services, Data Collection, Documentary Analysis, Managers, Health Professionals, Patients

## Abstract

**Background:**

The main purposes of primary care-based pharmaceutical services (PHCPS) in Brazil are to provide free access to medicines and pharmaceutical care to patients. Several obstacles hinder achieving their goals; thus, MedMinas Project aimed to evaluate the PHCPS, the supply system, and the use of medicines. This paper reflects on our experience designing, planning, and conducting the project, describing the issues yielded in the field and lessons learned.

**Methods:**

This work consists of a mixed-methods study conducted in Minas Gerais, Southeastern Brazil. We adopted the principles of Rapid Evaluation Methods, employing a multistage stratified sampling for the quantitative and a purposeful sampling for the qualitative components, respectively, and a documentary research. Data sources included individuals (patients and professionals), prescriptions, dispensed medicines, and policy documents collected between April and October 2019. The quantitative data described in this paper were analysed by descriptive statistics and the qualitative by Thematic Content Analysis.

**Results:**

A total of 26 municipalities varying from 37,784 to 409,341 inhabitants were included. The field team spent, on average, 16 days in each location. We interviewed 1019 respondents, of which 127 were professionals and 892 patients. The participation rate varied from 92 to 100%, depending on the respondent subgroup. Most interviews lasted between 45 min and one hour. Fieldwork challenges included participants’ enrolment, field team, interview processes, and project budget. The participants provided positive feedback and five main themes emerged from the interview experience (self-awareness, sense of gratitude, research value, access to findings, and benefits of the research). Additionally, we collected copies of 1072 documents and 2070 pieces of data from prescriptions filled and medicines dispensed at the PCP.

**Conclusion:**

We demonstrated the viability of conducting the MedMinas Project in an extensive geographic area within effective time frames that provided meaningful, high-quality data from multiple actors. The methods and lessons learned are valuable for researchers across various disciplines in similar urban settings in Brazil and other countries of low- and middle-income (LMIC).

## Background

Strengthening primary health care has been a priority for the Brazilian government in the designing and implementation of policies and funding [1, 2]. Part of the country’s strategic policies refer to the Primary care-based pharmaceutical services – PHCPS program, launched in 1998 as an essential component of the National Medicines Policy [3, 4].

The PHCPS programme is part of the National Healthcare System in Brazil (Sistema Único de Saúde—SUS), tax-funded and universally accessible to all Brazilian citizens, free of charge. The Ministry of Health and States Health Secretariats have roles in coordinating and regulating policies and actions, and, as the health system is decentralized, program delivery is under the responsibility of the Brazilian municipalities (*n* = 5570). The local program network includes the Municipal Health Secretariats and public community pharmacies (PCP). PCP cover specific territories and populations, offering treatment to a range of acute and chronic conditions, such as infectious and parasitic diseases, hypertension, diabetes, and chronic respiratory conditions [1].

The PHCPS programme is structured into two main subsystems: the management subsystem and the pharmaceutical care subsystem. The main goals are to provide free access to quality and cost-effective medicines, ensure adequate dispensing practices, contribute to the safe and effective use of prescribed medicines by patients, and conduct health-promoting interventions both individually and collectively [5]. However, the programme is still underdeveloped in Brazil, even existing for more than 20 years.

The implementation and performance of the PHCPS are subjected to different interests and contexts, thus its outcomes vary significantly across the territories. Overall, management styles are usually guided by bureaucratic bias, jeopardizing the intended goals [6]. Several challenges persist, such as poor facilities’ infrastructure and organization [7–9], medicine shortages and stock-outs at PCP [10, 11], and inadequacies in medicine storage and inventory control [12]. Additionally, a large number of patients do not know how to take their prescribed treatment properly [13].

We proposed the MedMinas Project to contribute to overcoming these challenges. The main purpose was to evaluate the PHCPS programme delivery, the supply subsystem, and the use of medicines by patients, examining processes and contextual factors to understand how the programme operates and identify improvement opportunities.

The MedMinas Project began in 2018 and ended in 2021 and involved planning and designing, fieldwork preparation, and data collection, management, and analysis. This paper aims to describe the study design and methods and to reflect upon the fieldwork experiences and lessons learned while conducting this project.

## Methods

### Research framework

The development of the MedMinas research framework required a targeted literature review concerning primary care and pharmaceutical services: (1) Brazilian national and state-level policies, and both national and international guidelines [5, 14–30]; (2) relevant methods and findings from selected quantitative and qualitative research, considering Brazilian and international scenarios [3, 7, 31–33].

We adopted a system-thinking approach to include different actors, their interrelations, and PHCPS functions [34]. Thus, the next step was guided by defining the scope, concepts, and measurement domains for each essential dimension, covering aspects of the triad “structure–process–outcome”, proposed by Donabedian [35]. We included data related to professional evidence, such as practice experiences and workforce satisfaction. We also incorporated contextual evidence, such as local policies and guidelines and individual factors (e.g., socio-demographic, economic, geographical, environmental, and epidemiological variables), considering their importance for health service planning, management, delivery, and outcomes [30, 31].

**Fig. 1:**
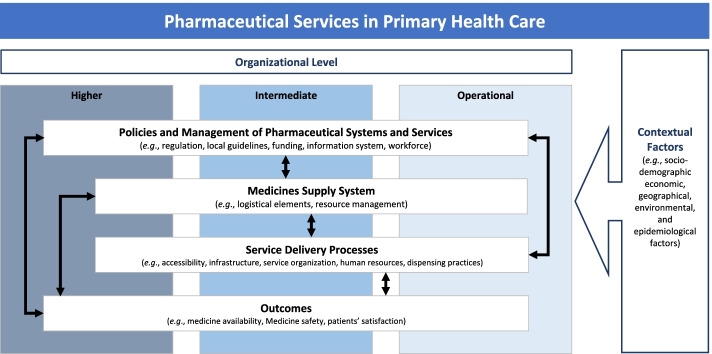
MedMinas logic model. Organizational Level (Municipal Health Secretary); Intermediate (Director of Primary Health Care Services and Municipal Coordinator of Pharmaceutical Services); Operational (Pharmacist and Dispensary Assistant)

The final framework is represented in Fig. 1. We incorporated three healthcare system organizational levels: 1) higher (Municipal Health Secretary); 2) intermediate (Director of Primary Health Care Services and Municipal Coordinator of Pharmaceutical Services); and 3) operational (pharmacist and dispensary assistant). The performance of the PHCPS system is viewed in terms of its outcomes (medicine availability, medicine safety, patient satisfaction) and in terms of related and underlying structures and processes that explain these outcomes and their interconnectivity, such as public policies and management of pharmaceutical systems and services, medicine supply system, and service delivery processes. Contextual factors involved variables related to patients and the environment.

### Study design and setting

The MedMinas Project has multifaceted research questions, both qualitative and quantitative. Therefore, we adopted a mixed-methods study design based on Rapid Evaluation Methods (REM) [36–38]. REM are useful to appraise the quality of health care services, identify problems, and guide managerial responses [39].

The study area was the State of Minas Gerais, located in Southeastern Brazil, the fourth largest State in the country, with a territorial extension of 586,528.293 km^2^. Minas Gerais was purposefully selected for this project for being the largest State concerning the number of municipalities (*n* = 853), the second-most populous (20,997,560), and the third by gross domestic product [40, 41]. The State is considered high in HDI (0.731) and ranks 9^th^ among the 27 Brazilian states [42]. Minas Gerais is divided into 13 macro-regions (South, Central-South, Centre, Jequitinhonha, West, East, Southeast, North, Northwest, Eastern South, Northeast, South Triangle, North Triangle) and 76 micro-regions according to its Regionalisation Directing Plan [43]. Following the principles of decentralization of the health system, each of these regions has health pole municipalities, serving as healthcare references to other cities, i.e., they are considered healthcare standards in terms of equipment, human resources capabilities, and service provision to the other municipalities in the same region [21, 43].

The overall health profile of Minas Gerais shows a strong relative predominance of non-communicable diseases, such as cardiovascular conditions, cancer, and respiratory diseases, which are responsible for 66% of the disease burden, coexisting with infectious and parasitic diseases, road injuries, and interpersonal violence [44]. However, the mortality and morbidity profiles vary across State regions due to differences in the micro-political, economic, and socio-cultural environments [45].

The MedMinas project was designed under three premises: (1) fifty percent of the population of Minas Gerais (10,6 M inhabitants) live in medium to large-sized municipalities (between 35,000 and 900,000 inhabitants); (2) the healthcare management and organization model adopted in the State follows the Minas Gerais Regionalisation Directing Plan [43]; (3) evidence suggest suboptimal PHCPS performance in large municipalities, highlighting the need of specific assessment targeting these sites [46].

### Information and data sources

Four types of information and data sources were included: (1) patients and health staff from PCP related to Primary Care, (2) health managers, (3) prescriptions and dispensed medicines, and (4) policy documents.

## Sampling

### Quantitative component

A multistage sampling technique was used to select the PCP, combining REM sampling recommendations of at least 20 health care facilities and 30 patients in each one, plus population size and geographic location criteria (Table 1). The first stage was selecting the macroregions of Minas Gerais, the second, the selection of municipalities within each macroregion, the third stage involved the selection of the PCP, and the fourth, patient selection in the facilities, as follows.

- **Stage 1.** All 13 macroregions were included, a priori, in the sampling plan to ensure representativeness. The number of municipalities to be investigated in each region was estimated in proportion to their population. Percent fractions were rounded up to the nearest whole number, so the final sample size was 26 municipalities.
- **Stage 2.** This stage comprised the selection of the municipalities within each macroregion. Eligible municipalities were those rated as health poles in the Minas Gerais Regionalisation Directing Plan [43] and with a population size ranging from 35,000 to 900,000 inhabitants (medium to large size-sized municipalities), totalling 68 municipalities. Formal invitations were sent, and those who agreed were included in the study, respecting the criteria established in stage 1.
- **Stage 3.** This stage involved the selection of public community pharmacies (PCP). One PCP was selected from each municipality. The main advantage of this method is that experiences with similar methodologies have shown that treatment practices of an individual health care provider are similar to the set of providers with the same resources, so variations in the same services tend to be reduced within each municipality [47]. Thus, the selection of the PCP in municipalities with more than one facility was based on the proximity of the Municipal Health Secretary to reduce logistic costs.
- **Stage 4.** The final stage was the selection of patients. According to REM, we randomly selected patients to be interviewed immediately after the contact with the PCP to assess the functioning of the health care facility from their perspective. To estimate the patient sample, we considered the number of PCP to be visited in each macroregion and the minimum number of 30 patients to be interviewed in each service [36]. A percentage of 20% was added to this sample to compensate for losses, totalling 936 patients (Table1). We included a random selection of patients aged 18 years or older who were patients from the PCP for at least six months and had their prescription and dispensed medicines in hand.

**Table 1:** Sampling process. MedMinas Project, 2018–2021

Macroregion	Population		Number of selected municipalities	Minimum number to be included per macroregion			
	***n***	**%**		**PCP**	**Patient**	**Managers**	**Health professionals**
Centre	6,574,968	31.3	7	7	252	14	14
South	2,787,614	13.3	3	3	108	6	6
North	1,678,958	8.0	2	2	72	4	4
Southeast	1,669,802	8.0	2	2	72	4	4
East	1,536,591	7.3	2	2	72	4	4
North Triangle	1,281,989	6.1	2	2	72	4	4
West	1,276,557	6.1	2	2	72	4	4
Northeast	935,587	4.5	1	1	36	2	2
Central-South	788,353	3.8	1	1	36	2	2
South Triangle	768,771	3.7	1	1	36	2	2
Northwest	699,974	3.3	1	1	36	2	2
Eastern South	699,751	3.3	1	1	36	2	2
Jequitinhonha	298,645	1.4	1	1	36	2	2
**TOTAL**	20,997,560	**100**	**26**	**26**	**936**	**52**	**52**

*PCP* Public Community Pharmacies

### Qualitative component

A purposeful sample of two subgroups of health professionals was used to select key informants in each of the 26 municipalities included in the quantitative sample to capture potentially rich information [48]. We first undertook a desk review, mapping out managers responsible for outlining strategies and actions for Primary Care at the municipal level. Three levels of healthcare system managers were included: the Municipal Health Secretary (1), the Director/Coordinator of Primary Health Care Services (1), and the Municipal Coordinator of Pharmaceutical Services (1). The second group of professionals consisted of representatives of the PHCPS staff: a pharmacist (1) and a dispensary assistant (1). Thus, the estimated sample totalled five professionals per municipality, 130 considering all the 26 municipalities investigated. Considering the possibility of refusals, we sought to include at least two representatives of the subgroup of managers and two representatives of the PCP. Another municipality filling the correspondent inclusion criteria was selected in the case of refusals from more than one manager. If possible, in case of refusal of PCP staff, another facility from the same municipality was selected. Otherwise, the municipality was replaced.

### Documentary research

In the Brazilian National Health System (SUS – acronym in Portuguese), some policies and instruments should be used for planning and management. Considering the relevance to primary care and PHCPS, several documents were selected for analysis and collected during the fieldwork: Municipal Health Plan (*Plano Municipal de Saúde*), Municipal Essential Medicines List (REMUME – *Relação Municipal de Medicamentos Essenciais*), collected at the Municipal Health Secretariat, and copies of prescriptions filled at PHCPS.

We also included the Annual Management Report to be extracted from the SARGSUS database (*Sistema de Apoio à Construção do Relatório de Gestão*). This database was developed by the Ministry of Health and contains, among other information, the results of PHCPS achieved during the execution of the Municipal Health Planning.

### Preparation for data collection

#### Research team

The MedMinas research team comprised three groups of members: general coordination, research staff, and field team. General coordination was composed of four members, the principal investigator, the co-principal investigator, and two field coordinators. This team was responsible for, among other duties, assuring study protocol compliance, developing and maintaining records of all the study documentation, recruiting, training, and managing the field team, managing the data collection process, and storage of research products.

The research staff was composed of one administrator and five students (four graduates and one undergraduate). The administrator was responsible for maintaining diaries, arranging appointments, handling correspondence, and following up with target participants, while the students participated in developing part of the study documentation and data collection.

The field team comprised three statisticians, two field supervisors, three field support personnel, and twenty interviewers. This team was assembled by a third party hired by the general coordination and assigned to collect data and prepare field registers and databases.

### Documents and materials

The general coordination and research staff developed the MedMinas logo representing the project’s visual identity to customize digital and printed documents and materials (e.g., slides, letters, certificates, banners). Communication guides and letters and electronic correspondence templates were developed to improve recruitment and stimulate participation in the study. We developed comprehensive and detailed manuals to guide the field team, informed consent to each participant profile, a graphic scale for collecting patient data, and leaflets and banners containing concise information about the project to support the recruitment process. Considering the diverse profile of the MedMinas target audience, we also prepared communication guidelines and letter and electronic correspondence templates. Furthermore, we prepared written information about the relevance of the research, its implications, and advances in science.

We also developed two health education materials to be distributed to participants during data collection: (1) a health calendar, designed to help personal management of healthcare appointments, containing a schedule card with relevant information about the Brazilian adult immunization programme; and (2) a booklet covering aspects of the correct use of medicines and information about medicine storage.

### Instruments

The main purpose of the instruments was to operationalize concepts and domains from the study framework and explore multiple individual perspectives while enabling result triangulation. Accordingly, specific questionnaires were developed for the following respondent profiles: patients (1), dispensary assistants (1), pharmacists (1), Municipal Health Secretary and Directors of Primary Health Care Services (1), and the Municipal Coordinator of Pharmaceutical Services (1).

Instruments development was conducted by general coordination and involved four steps. The first step (1) consisted of listing the main variables of the study, searching the literature [49–54], and reviewing previously tested instruments used by our research group in similar investigations [7, 10, 13, 55, 56]. This step allowed us to focus on concepts that should be measured or operationalized during data collection, considering our target audience, characteristics, and potential limitations. Step (2) consisted of consulting a panel of specialists to discuss content, format, quantity, complexity, level of detail, time sequence (present, past, future), logical sequence of the questions, and type of question (tick box, date, Likert scale, date, short-free text and open-ended). Step (3) involved elaborating the preliminary version of the instruments. Five instruments were developed, formatted, and edited to be applied by electronic devices. The instruments ended with the following closing statement to collect participant feedback: “We have reached the end of our interview. We would like to thank you for your participation and ask you to register your last impressions or thoughts. Do you have any additional comments or suggestions?”.

A series of pretesting was conducted in Belo Horizonte, the state capital of Minas Gerais and headquarters of our research institution. The procedures involved the research staff, the field team, and academic and non-academic volunteers with a similar profile to the study population. We evaluated the performance of each instrument in terms of comprehensiveness, clarity, the flow of questions, timing, and the recording process. Step (4) consisted of obtaining the final version of the instruments. Domains and main topics explored in the instruments are presented in Table 2. The level of detail varied according to the job position in the healthcare system, health managers and health professionals.

**Table 2:** Questionnaires by domain, topic, and participant subgroups. MedMinas Project, 2018–2021

Subgroup	Questionnaires	
	**Domain**	**Topic**
**Health managers and health professionals**	Pharmaceutical Services at Municipal level	Local policies, management, and evaluation of pharmaceutical services, governance and leadership, information systems, workforce, forecasting and medicines procurement, availability of medicines, and medicines coverage
	Perceptions on PHCPS	Service delivery, facilities infrastructure and organization, staff (number, professional training, interpersonal relationship, and working processes), and dispensing practices
	Socio-demographic data	Age, gender, job position and time on the job, and professional training
**Patients**	Social Capital	Perceptions of trust, safety, help, social support, and community integration
	Health status and health-related behaviors	Self-rated health, past medical history, alcohol consumption, smoking, and use of medicines
	Access and use of health care/PHCPS	Use of health services in general and PHCPS, perceptions on PHCPS (service delivery interaction with the staff, facility infrastructure and organization, geographic access, effectiveness, and level of satisfaction), prescribed and dispensed medicines, and private spending patterns on prescribed medicines
	Medicine safety	Knowledge of prescribed medicines, household storage, and disposal
	Socio-demographic and economic data	Age, gender, skin color/race/ethnicity, marital status, educational level, income, private health insurance, and household composition

All instruments were formatted for tablet-based data collection using the software SurveyToGo^©^. A data validation process was conducted to verify data entry, review instructions, test skips logic, constrain responses when applicable, and evaluate potential errors and inconsistencies. Additionally, there was the last round workshop involving the research team to evaluate questions that needed rewording or that should be removed from the instruments.

### Training

In order to ensure high-quality data collection, the general coordination prepared and delivered a 90-h training course for the field team, covering practical knowledge on field research, epidemiology, qualitative research, ethics, interviewing, and providing project-specific knowledge, especially regarding collecting accurate data on medicines and prescriptions.

The training course comprised a variety of classes, both in group and one-on-one lessons. Slide presentations, video classes, and a workbook were provided. Trainees were tested and granted certificates. Training materials were stored in a cloud service and thus accessible to all the research team members regardless of location.

During training, for ethical reasons and safety issues, interviewers were strongly encouraged to place project materials and electronic devices securely and out of sight in the project bag when not in use.

### Enrolment

Two complementary strategies for enrolment were developed, one for municipalities and another for health managers and professionals.

First, a database containing relevant information from 68 eligible municipalities, such as contact details of the key informants and Municipal Health Secretariats and PCP, was prepared. To minimize selection bias, all the eligible municipalities, in the person of the Municipal Health Secretariat or a representative, were contacted by standardized telephone calls and formal written invitations (electronically and by mail). We obtained a low response rate using this strategy, so we asked the State Health Secretariat to formally present the project to managers and representatives of Municipal Health Secretariats.

Our presentation was delivered at the state-level collegiate instance of SUS, the Bipartite Inter-managers Commission. We then sent new invitations to the eligible municipalities, and those that agreed have provided letters of acceptance. Managers and health professionals from those municipalities were then contacted by telephone and email to be informed about the project details and confirm their individual acceptance to participate in the study. We included municipalities where at least two managers and two health professionals agreed to participate in the study.

The municipalities and participants entered the study according to a process of first-served basis that ended when the sample size quotient of 26 municipalities was full.

### Data analysis

This paper describes the results of our experiences conducting the pilot study and data collection processes, reflecting upon the lessons learned. We used descriptive statistics for quantitative data. Thematic Content Analysis [48] was used to analyse respondent reflections upon the interview experience. We included all managers (*n* = 77), health professionals (*n* = 50), and a random sample of 10% of the patients (*n* = 83). Microsoft Excel® (Microsoft 365 MSO) was used for the quantitative and qualitative analysis.

## Results

### Pilot study

The pilot study was conducted from February to March 2019 and included a sample of patients and one health professional (*n* = 31) in five municipalities. This study had the purpose of increasing knowledge and assessing the practicalities of the field, highlighting aspects that could potentially compromise data collection [57]. We identified problems related to the field team, equipment, research participants, and venues.

The main problems regarding the field team were the interviewers’ lack of experience. Errors were committed when interviewing patients that did not fill all the study’s inclusion criteria; ignoring the ethical rule that requires participants to sign the informed consent before the interviews; taking blurry pictures, conducting interviews in noisy places, reading questions differently from the original text, and in entering wrong participant answers. Regarding equipment, we detected problems in recording and uploading the interviews. Some participants and study sites – especially the PCPs – offered extra challenges to data collection, such as asking for the research instruments in advance, trying to eavesdrop or interfere in other participant interviews, complaining about the presence of the field team. Some of the PCP did not have the minimal infrastructure for conducting research, i.e., room arrangements and furniture.

After the pilot study, another round of contact by telephone and email with the municipalities was conducted to clarify the study purposes and methods and arrange data collection on the venues. We also invested in a focused refresher course to upskill the field team. We took pictures of prescriptions and medicines during interviews to increase the quality of data collected from these items.

The technicalities were solved, and a protocol for field monitoring and auditing was developed by the general coordination team, including criteria for interview approvals and details on in-process quality verifications, such as listening to recorded interviews and database review.

### Data collection

**Fig. 2:**
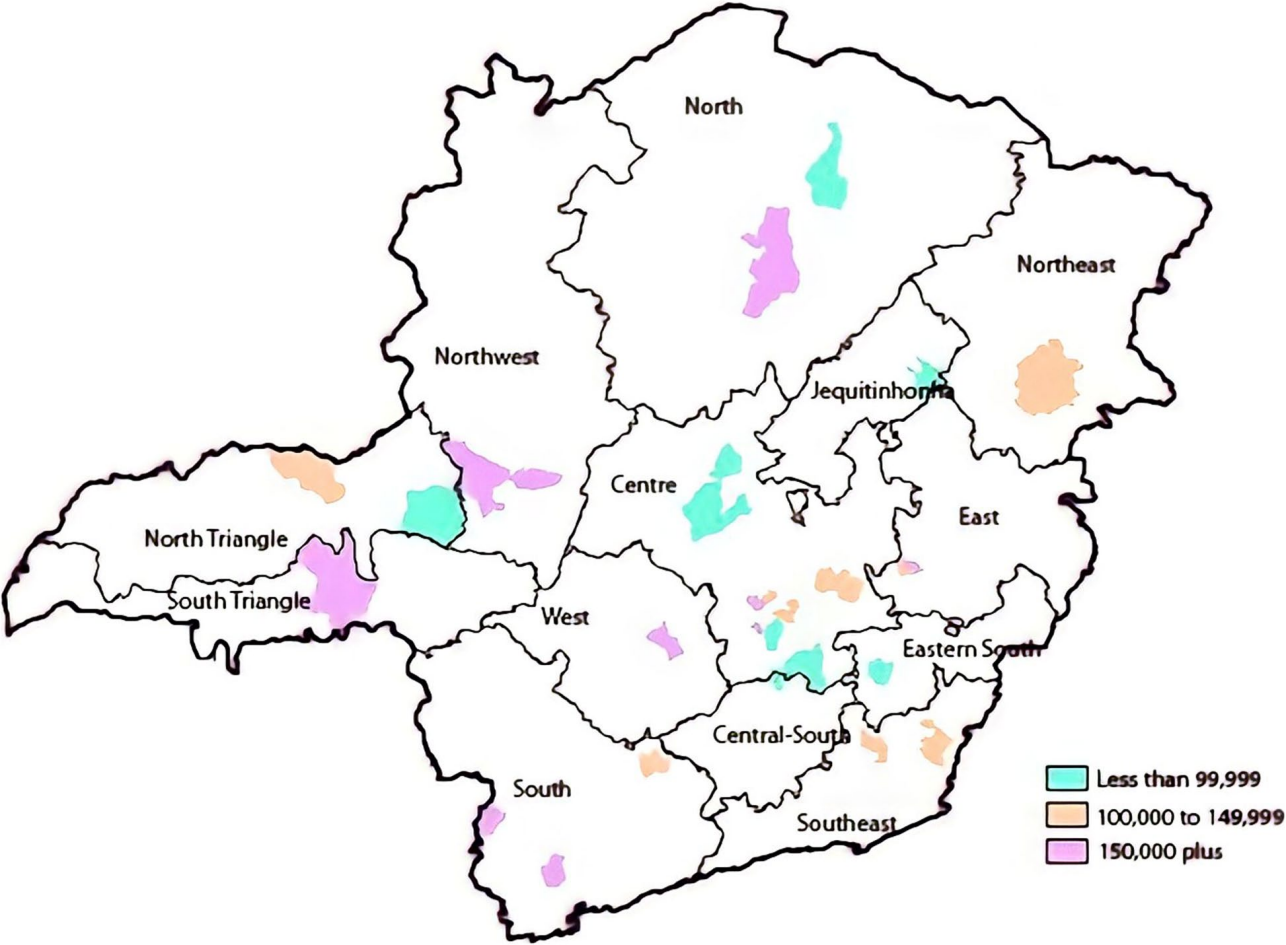
Map of the macroregions of Minas Gerais and the municipalities included in the MedMinas Project by population size

A total of 26 municipalities were included in the project, and none of them withdrew the acceptance after the study began (Fig. 2).

Data was collected from April to October 2019 and began by the municipalities located at the centre macroregion due to the proximity to the project’s leading institution based in Belo Horizonte, where the coordination team is based.

The field team was gradually sent, either in pairs or solo, to each of the 26 selected municipalities, following a standardized data collection plan and detailed daily schedule. Upon arrival in each municipality, the field team delivered introductory letters to health professionals at the selected facilities and health managers at the Municipal Health Secretariat, even though we had a formal agreement with all the municipalities and had exchanged comprehensive information about the project with health managers and professionals before going to the field. Only those that agreed to participate were interviewed at the workplace.

Most managers and professionals were fully adherent to the project. One Municipal Health Secretary refused to participate, and another asked to be replaced by the Municipal Director of Health Care, which is why the subgroup of “Coordinators of Primary Health Care” is over-represented (Table 3). Additionally, the Municipal Coordinators of Pharmaceutical Services of two municipalities were simultaneously responsible for the PCP investigated, reducing the number of pharmacists interviewed by 2.

**Table 3:** Respondent groups by the total number of participants, selected demographics, and participation rate and interview duration. MedMinas Project, 2018–2021

Respondent Group		Total number of participants (n) and participation rate (%)	Demographics			INTERVIEWS	
						**Average Duration (min)**	**Total Duration (hours)**
			Mean Age (SD)	Female Sex n (%)	Education (≥ 14 years of study)		
MUNICIPAL MANAGERS	Health Secretary	24 (92.0)	48.6 (11.2)	14 (58.3)	24 (100.0)	00:50:53	20:21:14
	Primary Care Coordinators	27 (103.8)	39.2 (9.1)	24 (88.9)	27 (100.0)	00:42:46	19:14:47
	Pharmaceutical Services Coordinators	26 (100.0)	41.0 (10.0)	16 (61.5)	25 (96.2)	01:27:47	38:02:10
PCP HEALTH WORKERS	Pharmacist	24 (92.0)	35.8 (6.9)	18 (75.0)	24 (100.0)	01:09:37	27:50:44
	Dispensary assistants	26 (100)	37.9 (11.8)	22 (84.6)	10 (38.5)	00:53:46	23:18:06
PCP USERS	Patients	892 (95.3)	53.0 (15.5)	561 (62.9)	73 (8.2)	00:56:26	835:19:59
						**TOTAL**	964:07:00

Patients were recruited at the PCP. Before dispensing, potential participants were approached by the interviewers, who introduced themselves, distributed the project leaflets, and informed about the general purpose of the study. Patients were approached again after dispensation. Those meeting the inclusion criteria were invited to participate while being fully informed about the study’s purpose, risks, benefits, and interview duration to make a conscious decision to accept or decline participation. These procedures were adopted to minimize selection bias and guarantee a probabilistic sample.

We interviewed 1019 respondents, of which 127 were professionals and 892 patients (Table 3). The participation rate in the study varied from 92 to 100%, according to the respondent subgroup. The mean number of interviews per municipality was 39 (SD ± 7). We achieved 97% of the maximum sample planned for professionals and 95.3% for patients. Most participants were female. The mean age varied from 35 to 53 years, depending on the participants' group. In general, patients were older than managers and health professionals. Most managers and health professionals have attained tertiary education, while the same rate among patients was minimal (8.2%).

We recorded almost 1000 h of interviews (Table 3), most of which lasted between 45 min and one hour. PHCPS coordinators went through the lengthier interview processes (mean time of 1 h 28 m).

The field team spent, on average, 16 days (SD ± 4.9; min, max: 8–24 days) in each location. Despite contacting municipalities ahead of visiting, data collection took more days than planned due to the non-availability of patients to participate in the study caused by medicine shortages at the PCP. In one municipality, patient recruitment was not feasible, so we lost patient data, while in another, we had to change data collection to another PCP facility because the patient flow was minimum.

We collected copies of 25 Essential Medicines Lists (REMUMEs), 26 Municipal Health Plans, 26 Annual Management Reports, and 995 prescriptions during the fieldwork, totaling 1072 documents. Additionally, we took 2070 pictures of prescriptions filled and medicines dispensed at the PCP.

### Data monitoring and management

The field supervisor managed interviewers during the entire data collection process to ensure adherence to the study protocol, data quality, and completeness of the fieldwork. The team also inspected documents and images collected in the field. The coordination team oversaw the entire data collection process, assisting the field team in real-time and conducting in-process quality checks.

Additionally, we conducted a thorough auditing process for the first ten municipalities (*n* = 365) and a random sample of 10% of the other 16 municipalities (*n* = 70), totaling 435 interviews (42% of all interviews). The audit protocol included reading through all the questionnaires and listening to the correspondent audio-based records in detail to identify and correct errors or inconsistencies in data entry. These procedures were also used to evaluate interviewer performance, such as remaining neutral during interviews while establishing empathy and rapport with interviewees, asking questions correctly, and clarifying the questions.

During the auditing process, we found that around 24% of patient interviews contained minor errors and a few inaccuracies, especially regarding information collected from prescriptions and dispensed medicines. The interview software, for instance, was programmed to collect data from one prescription at a time, but some interviewers, especially at the beginning of the fieldwork, included information from more than one prescription together as if they were just one document. Another problem was data entry as “unreadable” or “missing”, when, in fact, we could identify the correspondent information in the prescription or in the dispensed medicine by checking photos taken during the fieldwork. We produced a report based on these problems and discussed them with the field team.

Data management included the storage in electronic folders and backup copies of all field products (questionnaires, documents, images, audio-records) organized by municipality and respondent profile. All field products were protected by passwords with access only to the coordination team.

Since MedMinas instruments contained both open-ended and closed-ended questions, we transcribed the interviews. These procedures began after completing the fieldwork due to a previous agreement with the third party assigned to collect data that had internal procedures to prepare and deliver the field products.

### Fieldwork challenges, lessons learned, and participant feedback

MedMinas had complex field logistics addressing transportation, accommodation, field materials, and equipment. The most significant fieldwork challenges concerned: (1) municipality and participant enrolment; (2) field team; (3) interview processes; and (4) project budget. Table 4 summarises the occurrences we faced on the ground and the lessons learned from these challenges.

**Table 4:** Fieldwork challenges and lessons learned. MedMinas Project, 2018–2021

Fieldwork challenges	Occurrences in the ground	Lessons learned
Municipalities and participant enrolment	Our first attempt to enrol them was unsuccessful, even providing information and high-quality field materials for eligible municipalities Despite having previously agreed, some managers and health professionals, when face-to-face with our field team, re-scheduled or refused to participate in the study	Obtaining upfront support and establishing liaisons with relevant key health system actors is crucial to increasing research engagement. Our strategy of involving the state-level collegiate instance of SUS was pivotal Extensive contact made by the general coordination with the potential participants, explaining the study goals and applicability of results thoroughly and making the team available to them before and during the entire fieldwork, improved the participation rate, especially for managers and health workers since the target sample was fixed
Field team	Providing appropriate individualized and collective training for data collection could not prevent errors and inconsistencies in data entry during the fieldwork, especially regarding prescriptions and dispensed medicines	Recruiting a field team with appropriate skill sets is critical. Most problems could be avoided if the team had a background in pharmacy or related sciences
Interview processes	We knew that the sites of the interviews in the municipalities could be a challenge due to their infrastructure, so we asked managers and health professionals for help in choosing a setting with as little distraction as possible. However, in practice, some interviews got interrupted due to other work demands/competing priorities of the participants	Interviews in the workplace should be conducted in a neutral environment. This can be done by booking rooms separated from professionals’ offices (if possible, in another facility) or scheduling interviews after working hours
Budget	Fieldwork was planned with a reasoned time frame, drawn up via our previous experience conducting similar projects and based on the literature. However, data collection took longer than expected due to medicine stock-outs resulting in additional unanticipated costs	When conducting projects with complex logistics, one must consider the possibility of delays in data collection that will impact the estimated budget. We had the opportunity of additional funding to support the project, but this is not always a possible solution. Budgetary and managerial flexibility must, then, be considered during planning

Regarding participant feedback, 39% of managers (*n* = 30), 60% of the health workers (*n* = 30), and 70% of patients (*n* = 57) informed that they did not want to add thoughts or impressions about the interview. However, even without further elaboration, several of them mentioned the interview covered everything they expected or declared *“I am good”*, *“I cannot think of anything else right now”*, “*I have nothing else to add*”.

On the other hand, most managers (*n* = 47; 61%), and around a third of health workers (*n* = 18; 36%) and patients (*n* = 26; 31%), broadly reflected on the experience of being interviewed. Five common major themes emerged from these groups: *“self-awareness”, “sense of gratitude”, “research value”, “access to findings”*, and “*benefits of the research”*. Numerous participants mentioned more than one theme.

Self-awareness was the predominant theme (*n* = 50, 46.3%), meaning that the process of being interviewed has encouraged respondents to think about issues that they had not previously thought about:

“I am glad that you came here to my city! The interview made us reflect upon many things, even about the work we've been developing here, right? We're so in this fire-drill mode that there is no time to stop and plan the work that has to be done” (Coordinator of Pharmaceutical Services, 7)

“This interview has already yielded results. I will set up a meeting with the Municipal Coordinator of Pharmaceutical Services to create our ‘Pharmacy and Therapeutics Committee’ and to update our municipal medicines list (sarg)… All the flaws I've mentioned here… Emm, this interview really gave me the opportunity to do something about it!” (Health Secretary, 16)

“I think that health professionals should be more valued, and more jobs should be offered to them.” (Patient, 315)

“I wish they had more medicines available. There are too much stock outs… physicians prescribe the most expensive medicines, and they [the pharmacy] don't have it!” (Patient, 164)

A “*sense of gratitude*” for the opportunity of being heard also emerged (*n* = 23; 21.3%):

“Thank you for picking us. We know the project only included a sample of the municipalities in the State, so we feel like we won an award… We needed to be included, to have the opportunity of giving our opinion!” (Coordinator of Pharmaceutical Services, 12)

“There are places where we feel abandoned. It's good to see someone looking out for us. Congratulations on the research initiative!” (Patient, 467)

The “*research value*”, i.e., the relevance of the study, was pointed out by the participants (*n* = 15; 13.9%):

“I really enjoyed this interview… I really like this type of academic work, with the aim of ‘diagnosing’ our health system… I'm sure it will bring about benefit to us managers… Research is a powerful instrument.” (Municipal Health Secretary, 24)

“Extremely valid to understand what people think to improve [the pharmacies]” (Patient, 373)

The “benefits of the research”, i.e., a hope that the research findings will improve PHCPS, was considered by the interviewees (*n* = 11; 10.2%):

“I wish the results of this interview could benefit people here, especially pharmacy users. There are many sick people in our neighbourhood, even bedridden…” (Patient, 255)

“I hope this study makes it possible, right, for us, to do some effective change, right, in the Pharmaceutical Services in the city (Health Secretary, 2)”

Another key theme was “access to findings”, i.e., the importance of the participants being informed about the research findings. However, this theme emanated only from managers and health professionals (*n* = 9; 8.3%).

“I think the work you started by doing this investigation, if the municipality could be informed of this data collection results, it will contribute to our planning or even to the necessary adjustments. The health field is very dynamic, the challenges we face every day, we need to adapt to managing. People here need this outlook… other managers need this ‘outside perception’ you bring to us…” (Coordinator of Pharmaceutical Services, 25)

## Discussion

In Brazil, primary data collection is still essential for evaluating the performance of the healthcare system despite the existence of National Information Systems for more than 30 years [58]. Databases available in the country are remarkably limited, considering the need for information for planning, managing, and enhancing programmes such as the PHCPS.

We showed the viability of conducting a mixed-method study to provide relevant information on pharmaceutical services, medicine provision, and the use of medicine by patients, highlighting the diverse range of skills and attention to detail needed to take on complex projects of this nature.

Setting up this research project in one of the largest Brazilian States, Minas Gerais, proved to be a rich managerial experience. Considering this challenging research context, we knew from the beginning that the project would require unwavering preparation for data collection, and we would not obtain much knowledge from the literature. In fact, there is still no agreement on performance evaluation of primary care services [59] and even less information on PHCPS. Therefore, we developed the research tools, proposed the plan to achieve the goals, and established responsibilities and standards at an early stage.

The field logistics required much effort and was costly. We were benefited by obtaining an extra budget to complete data collection, but this is not a widespread reality in the research arena in Brazil, where there is a scarcity of potential funders for research focusing on the SUS, mostly coming from public sources [60].

We managed to ensure high-quality data despite challenges and unexpected on-the-spot difficulties from municipality and participant enrolment to the field staff, interview processes, and project budget. We believe the key element was balancing the efficiency of close monitoring with a degree of flexibility. Keeping the communication open and establishing connections and partnerships during the fieldwork helped improve the participation rate. The project adherence rates varied from 92 to 100% among the different groups of participants, which is very high. A nationwide study on PHCPS in Brazil, for example, reached participation rates of 61.5% for Municipal Health Managers, 84.5% for Coordinators of Pharmaceutical Services, 83.6% for health professionals responsible for dispensing medicines, and 97.8% for patients [61]. A study conducted in Minas Gerais reached participation rates of 85.6% for Coordinators of Pharmaceutical Services, 76.0% for health professionals responsible for dispensing medicines, and 92.4% for patients [46].

Another evidence that we could build trustworthy relationships on the field was the very positive feedback we had from participants. Many of them stated they were grateful for participating in the investigation, others recognizing that the experience of being interviewed has made them reflect on several issues related to PHCPS. Some participants happily indicated the relevance of the research, and some were hoping the results would help improve the PHCPS.

Some limitations of MedMinas must be recognized. A mixed-methods study, combining qualitative and quantitative approaches, is subject to selection and information biases and confounding. We minimized such interferences in all study phases. First, we developed a framework that considered PHCPS regarding its related underlying structures, processes, outcomes, contextual factors, and their interconnectivity. We operationalized the framework on multidimensional instruments, pre-tested and piloted, and applied them by a diligently trained field team that followed standardized procedures for data collection. We collected data from other sources, such as documents, prescriptions, and medicines dispensing. The characteristics of our patient sample are similar to the primary care population in Brazil [10, 62].

## Conclusions

The methods and lessons learned from conducting the MedMinas Project are useful and valuable for several reasons. We adopted universally applicable techniques suitable for researchers across various disciplines related to Public Health, enabling us to rapidly evaluate the PHCPS in an extensive geographic area and within effective time frames. Additionally, the very positive rapport we built with study participants and our data monitoring and management process allowed us to access meaningful data from multiple actors that we could not obtain otherwise. Finally, our experience may be applied in similar urban settings in Brazil and other LMIC countries.

## Data Availability

Quantitative data sharing is not applicable to this article as no datasets were generated or analysed during the current study. The qualitative data that supported this study are not public, but are available from the corresponding author on reasonable request.

## Abbreviations

HDI: Human Development Index
LMIC: Low- and Middle-Income Countries
MedMinas Project: Evaluation of pharmaceutical services, medicines provision and use of medicines
PC: Primary Care
PCP: Public community pharmacies
PHCPS: Pharmaceutical Services Based on Primary Care
REM: Rapid Evaluation Methods
REMUME: Municipal Essential Medicines List
SUS: National Health System
